# A Scoping Review: Autologous Fat Grafting to Improve Volume and Aesthetics of Cleft Lip Deformity

**DOI:** 10.7759/cureus.52632

**Published:** 2024-01-20

**Authors:** Kenneth Goich, Todd Schachter

**Affiliations:** 1 Medical School, Rowan-Virtua School of Osteopathic Medicine, Stratford, USA; 2 Department of Family Medicine, Rowan-Virtua School of Osteopathic Medicine, Stratford, USA

**Keywords:** coleman technique, adipose derived stem cells, autologous fat transplant, fat graft, cleft lip

## Abstract

A frequent problem following cleft lip repair is insufficient lip volume and unappealing aesthetics. Autologous fat grafting is a method of improving the appearance of post-correction deformity. The aim of this review is to evaluate the effectiveness of autologous fat grafting in improving the aesthetics of cleft lip deformity. The benefits of adipose-derived stem cells (ADSCs), benefits and complications of fat grafting, timing of grafting, and harvest and transplant techniques will be examined.

This review process used “PubMed” and “Google Scholar” as primary databases. Searches were performed using combinations of key terms: “Fat Graft,” “Cleft Lip,” “Vermillion,” “Autologous Fat Transplantation,” and “Adipocyte Derived Stem Cell.” Reviews of reference lists for additional pertinent data were performed.

Autologous fat grafting may be performed during primary repair or as a secondary correction. Statistically significant improvements in appearance were observed in some or all measured variables regardless of repair timing. Both timing options show favorable outcomes; however, there is more evidence in support of grafting as a secondary correction. Some degree of graft reabsorption will occur, lending evidence to the practice of overcorrecting to accommodate losses. Graft retention is stabilized by the 12-month mark. The presence of ADSCs within the graft aids in graft stabilization and retention. Despite a lack of longitudinal data to examine graft retention throughout a patient’s lifetime, autologous fat grafting appears to be a safe and minimally invasive method of repairing deformity secondary to cleft lip repair supported by follow-up data as far as two years postoperatively with minimal reported complications.

## Introduction and background

Cleft lip and palate are full-thickness defects that occur due to congenital malformations. These malformations are the result of the failure of fusion of the facial prominences during fetal development. This can lead to a variety of complications including feeding difficulties, ear infections and hearing loss, speech and language delay, and dental complications. As of June 2023, the CDC states that the incidence of cleft lip and palate occurs in ~1:1600 births and isolated cleft lip in ~1:2800 births [1].

Following surgical repair, patients born with cleft lip often lead healthy lives but may suffer from bullying, self-consciousness, depression, and anxiety secondary to their appearance following correction [2]. A person’s vision is naturally drawn toward asymmetry; therefore, any facial deformity is easily seen and can be a source of psychological distress. In addition to scarring, abnormal lip appearance post-repair may be due to insufficient lip volume following the reposition of skin and muscle [2]. There are numerous different approaches to the correction of superficial abnormalities following cleft lip repair. Some of the newer methods of reconstruction are CO_2_ laser ablation, local flap reconstruction, silicone gel sheeting, utilization of botulinum toxin, and fat grafting [3]. This review focuses on autologous fat grafting.

Autologous fat transplantation, or fat grafting, involves the aspiration of host adipose tissue from a selected site, processing of the collected fat, and finally, injection of the final product into the site that is being targeted by the procedure. It is already a well-established method used in many cosmetic procedures to improve appearance and tissue deformity [2]. This technique can also be utilized to improve the volume, contour, and tissue characteristics of the repaired cleft lip [2,3]. Ideally, a successful cleft lip repair will result in a symmetric nasolabial alignment with minimal scarring [2]. Symmetry and aesthetics are evaluated based on lip symmetry, the appearance of the vermillion border, and the profile of the upper lip. Autologous fat grafts contain adipose-derived stem cells (ADSCs), which may aid in improving the appearance of the repaired lip [4-8]. This transplantation can be performed during primary cleft lip repair or secondary to the original repair surgery.

**Figure 1:**
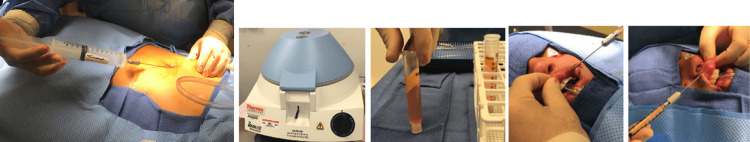
Tumescence of abdominal donor site, centrifuge and lipoaspirate, and fat graft from lateral intraoral injection sites These photographs are from [2].

The aim of this review is to evaluate the effectiveness of autologous fat grafting in improving the aesthetics of cleft lip deformity. Benefits of ADSCs, potential positive and negative aspects of fat grafting, timing of grafting, and harvest and transplant techniques will be examined.

Methods

Term Search Strategy

Peer-reviewed studies, published in English were retrieved. Data was collected from electronic databases (PubMed and Google Scholar) with searches performed using combinations of key terms: “Fat Graft,” “Cleft Lip,” “Vermillion,” “Autologous Fat Transplantation,” and “Adipocyte Derived Stem Cells.” This search was performed between September 19, 2022 and July 1, 2023. Reviews of reference lists for additional pertinent data were performed. Chosen studies examined adipocyte derived stem cells, primary and secondary fat grafting in cleft lip repair, fat harvesting, and grafting technique.

Data Extraction

All articles utilized for the purpose of this study consisted of scholarly papers found by the term search strategy and published between 2012 and 2022. Outcomes of identified articles were analyzed through quantitative and qualitative measures. Both measures were necessary to comprehensively determine the safety and efficacy of fat grafting, which involves the complex intermingling of subjective experiences as well as numerical data.

**Table 1:** Inclusion and exclusion criteria

Inclusion criteria
1. Full text available
2. Text is available in English
3. Published in peer-reviewed journal
4. Published during or after 2012
5. Case reports, literature reviews, and retrospective studies
6. Examines adipocyte-derived stem cells, primary and secondary fat grafting in cleft lip repair, fat harvesting, and fat grafting technique
7. Measurement of aesthetics or lip volume changes because of fat grafting
8. Target group is patients with cleft lip
Exclusion criteria
1. Full text unavailable
2. Text is unavailable in English
3. Not published in a peer-reviewed journal
4. Published prior to 2012
5. Not related to fat grafting as a method improving appearance in cleft lip repair
6. Does not measure changes in aesthetics or lip volume

## Review

Results

Adipose-Derived Stem Cells

When fat grafting, ADSCs, a component of the autologously harvested fat, have been shown to have positive effects on immune response and wound inflammation, promote angiogenesis, and reduce scar hyperplasia via collagen regulation [4-8]. Adipose tissue possesses an abundant amount of ADSCs in all age groups and can be collected in a safe, minimally invasive manner [6].

ADSCs are nearly identical to the stem cells collected from bone marrow and umbilical cord blood and possess similar differentiation capacities. There are some age-related differences; ADSCs collected from infant adipose tissue demonstrate greater angiogenic and osteogenic capabilities when compared to ADSCs from older adults [6].

Fat Grafting During Primary Cleft Repair

Fat grafting may occur during primary or secondary repair. A 2014 retrospective analysis by Balkin, Samra, and Steinbacher into the safety and efficacy of immediate fat grafting during primary cleft-lip repair showed that it can be done safely and may optimize results due to scar modulation and soft tissue augmentation. Thirty infants with a mean age of 3.5 months (aged 1.5-6.4 months) with a total of 37 sides (13 left, 10 right, and seven bilateral) underwent repair, and 20 of the 30 infants included in this analysis received fat grafting during primary repair [7]. An average of 1.4 cc (0.5-2.5 cc) was injected into the philtrum, piriform, vermillion, and ala, with all the injected fat having been collected from one or both thighs [7]. There were no reported complications at the surgical site nor the fat collection site, and the post-surgery mean follow-up was 24.7 months [7]. Photographs were provided to three blind reviewers who utilized a five-point ordinal scale rating the residual cleft-related facial stigmata (overall appearance of the full face, upper lip, nose, and midface) [7]. Each ordinal score (overall: 3:2.1; upper lip: 3.3:1.9; nose: 3.5:2.4; midface: 3:2.15) was statistically significant compared to those who did not receive primary fat grafting (p<0.05). Seven postoperative photographs showed minimal residual with inter-rater agreeability [7].

In a 2015 retrospective analysis of immediate fat grafting during primary cleft repair, 35 patients with a total of 44 sides (26 unilateral, nine bilateral) were examined [9]. Of these 35 patients, 20 underwent fat grafting along with the primary cleft lip repair while the remaining 15 patients served as a control for comparison [9]. The average age at surgery was 4.9 months [9]. Fat grafting occurred submucosally at the vermillion/mucosal junction, subcutaneously and intramuscularly along the scar/philtrum, and pre-periosteally at the piriform [9]. Three blind reviewers used photographs to grade patients on five-point ordinal scale to rate facial stigmata postoperatively, with results used for three different comparisons performed [9]. The first that compared overall results of the fat grafting group versus the control found statistically significant improvement (p<0.05) was appreciated in all areas, except around the nose, with inter-rater reliability scoring 0.78 on Cronbach alpha score [9]. The second results comparison examined only unilateral fat grafting group with unilateral control group subjects and found statistical significance in only face and upper lip ratings [9]. The final results comparison was between the 29 patients (16 experimental, 13 control) with greater than six months follow-up and showed the fat transplantation group had improvement in all aspects of the lip; however, only the upper lip improvements remained statistically significant when compared to the control [9].

Fat Grafting as Secondary Cleft Lip Repair

In 2017, Koonce et al. conducted a retrospective analysis that examined 52 children (mean age seven years) who underwent autologous fat transplantation as a secondary cleft repair between 2010 and 2016. All surgeries were performed by the same surgeon and all fat was collected from the abdomen or buttock with an average total volume of 3.0 mL used in the correction [2]. This fat was injected to correct the philtrum and vermillion border, as well as correct volume deficiencies in the vertical component of the lip with all patients receiving an overcorrection to account for fat reabsorption [2]. There were no reported complications with fat harvest or grafting with a mean follow-up time of 24 months and no patients required additional grafting [2]. Six blind reviewers graded pre- and postoperative photos on one-to-five-point scale, rating vermillion border, symmetry of the lip, and nasal profile including the upper lip, and when compared to the non-fat grafter patients, all values were scored significantly higher (p<0.05) with an inter-rater Cronbach alpha of 0.79 [2]. All patients and caregivers were pleased and stated they would have the procedure again [2].

Zheng et. al. performed a study including 65 patients who were treated by the same surgeon and received fat grafting to repair lip deformity secondary to cleft lip repair between January 2011 and January 2018. Fifty two patients had bilateral cleft lip with the remaining 13 patients having unilateral cleft lip [10]. The mean age at secondary repair was 22 years (range: 15-37) with mean age of fat injection at 25 years (range: 22-41 years) [10]. Donor fat sites included the thigh, abdomen, and buttocks with an average volume of 1.5 mL (range: 1-2 mL) injected into the philtrum, vertical component of the lip, and vermillion and all patients were over grafted by 0.5 mL to account for reabsorption [10]. Zheng et al. utilized a total of 12 reviewers, who consisted of a combination of males and females and included both lay people and experts in the field, to rate photographs of the original malformation and post-treatment. Symmetry and aesthetics were improved after fat grafting based on appearance of the vermillion border (P=0.02), symmetry of lip (P=0.007), and nasal profile including upper lip (P=0.04) using the Asher-McDade scale (one-to-five-point scale), with mean scale values scoring significantly higher (all P<0.05) when preoperative and postoperative values were compared (vermillion border, 3.1:4.17; symmetry of lip, 2.4:4.38; and nasal profile including upper lip, 3:4.21) with a Cronbach of 0.79 [10].

Jones et. al. examined 18 patients who received fat grafting as a secondary procedure following cleft lip repair. Grafting was performed between June 2006 and September 2012 [11]. The 18 involved patients had a mean of 16.1 years (range: 6-43), and of the sample, eight patients had bilateral cleft lip and remaining 10 had unilateral cleft lip, with all fat grafts collected from the periumbilical area and only one of the patients required a second fat graft despite overcorrection [11]. Jones et al. utilized two grading scales; the first of which was standardized preoperative and postoperative photos that were examined by two raters, a resident and an attending plastic surgeon. The second grading scale was a patient satisfaction questionnaire that was completed anonymously [11]. After grafting, all patients were rated as having a better appearance on the Asher-McDade scale with statistically significant improvements noted for the vermillion border (P=0.048) and nasal form (P=0.006) [11]. Of the 18 patients included in the study, 11 completed the satisfaction questionnaire and all rated the experience as positive with a statistically significant increase (P=0.001) in patient satisfaction regarding their appearance [11].

Bae et. al. examined 15 patients with a history of cleft lip repair who received autologous fat grafting to improve overall upper lip volume. Fat grafting was performed between November 2007 and March 2015 [12]. The patients had a mean age of 25 years (range: 17 to 55 years) and received a mean injection volume of 11.9 mL (range: 6.3 mL to 22.5 mL) of fat harvested from the abdomen [12]. Two methods of evaluation were utilized with the first being questionnaires administered to patients, plastic surgeons, and the general public [12]. Patients gave an average score of 3.80, plastic surgeons of 3.91, and the general public of 4.03, with all values showing that the volume increases were statistically significant [12]. The second method of evaluation involved measuring the changes in lip shape in pre- and postoperative photos, with results showing a mean increase of 46.71% (range: +9.59% to +121.43%) in upper lip protrusion, and a mean increase of 31.68% (range: -6.87% to +76.47%) in vermillion height [12]. All patients maintained the volume increase at the six month follow-up [12]. Bae et al. remarked that two of the patients in the study requested additional minor surgical revisions that were not planned at the time of fat grafting, and in these two patients the original engrafted fat was maintained at the two-year mark, even after additional surgery [12].

Whistle deformity has also been successfully treated with secondary fat grafting as well [2,13]. Baum et. al. examined the efficacy of autologous fat grafting in the correction of whistle deformity secondary to unilateral cleft lip repair in 15 patients with an average age of 21 years (range: 15 to 70 years). During this 4.5-year study, a total of 17 autologous fat grafts were performed with grafted fat being collected from the periumbilical area via liposuction for the grafts [13]. Thirteen out of 17 patients had a resorption rate greater than 50% and the overall mean was 53% resorption, but an overcorrection of only 30-40% was used to account for resorption and as a result, three patients were under corrected, two of which received a second transplant for further correction, the third declined further treatment [13]. When comparing the preoperative and postoperative ratios of vertical lip length, mean values were 0.87 and 0.93 (p=0.01), respectively, and 10 out 15 patients were satisfied with procedure at the six-month follow-up, and additionally those who received second grafting were satisfied with results following [13].

Fat Graft Survival

Restoring adequate blood supply to grafted fat is key to minimize reabsorption [10]. During the first four days postoperatively, the fat cells receive limited nutrition as the only means available are only via infiltration from surrounding tissue [10]. Long term survival is dependent upon revascularization of the graft, a process that takes a minimum of 48 hours [10]. Longer times without adequate blood supply increase the risks of necrosis, cyst formation, and fat absorption at the operative site [10]. The problem that arises is that the survival rate of fat cells is about four days, and if grafted fat is not revascularized in this time it will begin to become necrotic [10]. The value of using a fat graft rich in ADSCs was examined in an in vitro study by Eto et al. in which ADSCs were shown to be able to remain intact for 72 hours in ischemic conditions versus adipocytes that began to undergo necrosis or apoptosis within 24 hours and endothelial cells that began apoptosis in as early as 12 hours [14]. These findings were further supported by the evidence found in their in vivo study. Except for those located on the periphery of the graft, most adipocytes began to die on day one; however, by day three the number of proliferating cells had increased, and finally by day seven, a larger viable adipocyte area was observed [14]. This is suggestive of repair and regeneration in grafted fat [14]. The rate of vascularization and the density of formed vessels greatly influence survival of the graft. The lip area tends to have a rich blood supply. which may increase survival of fat graft [10].

Fat particles are intact globules of adipocytes and interconnecting mesenchyme [15]. Particle size of fat grafts may play a role in graft survival as well. Gause et al. states that en block excision of adipose tissue yields the largest particles, whereas when adipose is collected via liposuction cannulas, particle size is determined by cannula size, with larger cannulas yielding larger particles. Data as to the preferred fat collection method varies; however, one consistency remains in that the preference is for a larger bore cannula during collection because it leads less trauma and cellular rupture of collected adipose tissue [15]. A 2020 study on adipose particle in fat graft retention in rodent model found that the ideal particle size should be large enough to contain a sufficient amount of mesenchyme but not so large that the particle is unable to properly absorb nutrients [16]. Comparison between small- (2-4 mm) and large (5-7 mm)-sized particles resulted in similar graft retention. Even though larger particles underwent early hypoxia and adipocyte loss, the remaining tissue served as a framework for regeneration of new cells [16].

Due to somewhat unpredictable fat necrosis and reabsorption rates, plastic surgeons may opt to use for an overcorrection when fat grafting to account for losses to the area [2,10,11,13]. A 2017 analysis of 142 patients who received craniofacial fat grafting showed a significant (p<0.05), progressive graft retention during the first three postoperative months, with stabilization at 12 months with a mean 67.7% retention [17]. This average loss of 32.3% of grafted volume after 12 months lends support to surgeon preference to overcorrect when grafting.

Harvesting

There are a variety of techniques to harvest fat for grafting, with the goal of the graft being maximal graft survival rate and cell viability. When harvesting, the surgeon must make decisions on ideal donor site, harvesting procedures and cannula, and type of aspiration. A review by Fontes et al. showed possibly conflicting data on ideal donor site. Four studies, two using human fat grafted to animals and two examining patients who underwent fat grafting, showed no differences in graft survival regardless of collection site. However, another study in the review showed that the lower abdomen and inner thigh to contain the highest amount of ADSCs compared to other sites with this finding being supported in a second study that also found the outer thigh donor site to have the highest isolated fat graft or stromal vascular fraction (SVF) [18]. Finally, a comparison between superficial adipose tissue (SAT) and deep adipose tissue (DAT) showed SAT to be available in all fat sources in the body and to be associated with better stem properties making it an ideal donor site [18].

Fontes et al. states that there are a variety of procedures that can be used to harvest once a donor site is chosen, including direct excision, manual aspiration, and suction-assisted liposuction. If manual aspiration or suction-assisted liposuction are utilized, lower negative pressures are ideal, as higher pressures are more damaging to harvested adipocytes [18]. There is data suggesting no differences in cell viability between manual aspiration and suction-assisted liposuction [19]. The primary features of a cannula affecting its efficiency in fat collection are its diameter and number of holes [18]. Several studies have been performed comparing a number of different cannula sizes. These studies showed various comparisons between cannulas of different diameters including 2 mm, 3 mm, 4 mm, and 6 mm [18]. Each respective study showed more favorable outcomes with the larger cannula in the study [18]. Despite the lack of an agreed upon ideal cannula size, consensus is the diameter should be large enough to avoid shearing harvested cells [18]. In addition to collecting with low shear, injecting with low shear devices have been shown to increase graft survival and decrease lipolysis in humans and show increased retention in an animal model [19]. Once harvested, fat should be grafted as soon as possible to maximize survival [19]. Sammy et al. states that glycerol-3-phosphate dehydrogenase activity, a marker of adipocyte damage, rises linearly for four hours after harvest with harvested stem cells being able to survive for four hours at temperature or up to 24 hours at 4°C.

The final harvesting decision the surgeon must take is aspiration technique with the being to maximize adipose collected and minimize blood loss that may potentially make up a large portion of the aspiration. A variety of techniques have been proposed with some more effective than other for minimizing blood loss. Fontes et al. summarizes stating that dry technique has blood loss composing 20-50% of aspirate, wet technique with blood loss composing 4-30% of aspirate, super wet technique with blood loss composing 1-2% of aspirate, and finally tumescent technique with about 1% of aspirate being composed of blood. There had been concerns that using lidocaine as a local anesthetic could be damaging to fat cell; however, removal of lidocaine from lipoaspirate after collection corrected this issue [18,19]. Additionally, it has been shown that local anesthetics and vasoconstrictors have no significant effect on long term fat graft survival [18].

Once aspirated, there is a variety of ways to process the fat including centrifugation that allows for removal of the top oil layer and bottom blood and pellet layer leaving a concentrated fat sample, cotton gauze rolling to remove the oil and aqueous components, gravity separation with decantation and sedimentation allowing the lipoaspirate to separate overtime so the fat layer can be collected, and washing and filtering which may be done together or separate, involved washing with lactated ringers or normal saline whereas filtering utilizes a sterile metal sieve to isolate fat [20]. There is evidence in favor of all methods; however, no one method has been shown to be superior to the rest [19,20].

Coleman Technique

There are variety of techniques to perform fat grafting; however, the Coleman technique remains the most popular [21]. Harvesting in the Coleman technique involves infiltration of lidocaine with epinephrine via blunt tip 9-hole cannula at the donor site, then harvesting with a blunt tip 9-hole harvesting cannula with Luer-lock harvesting attached to a 10 cc syringe. Recommended harvest sites are the inner thighs, flank, or abdomen [22]. The aspirate is then refined by centrifuging for two minutes at 1286 g then removing the top and bottom layers. Finally, placement is performed by anesthetizing the site with lidocaine and epinephrine, then using a blunt Coleman cannula (type 1, 2, or 3) and injecting small amounts (max 0.1 cc) with each pass [22]. Postoperative dressing should not be utilized [22].

Discussion

In addition to function, an aesthetic appearance is one of the primary goals when correcting cleft lip deformity. Autologous fat grafting is one method of achieving this goal. The fat graft includes a variety of cells, most notable being the ADSCs. When fat graft is grafted it can be considered to be divided into three zones, peripherally is a surviving zone of adipocytes, followed by a regenerative zone of ADSCs that replace dying adipocytes, and a necrotic zone consisting of dead adipocytes and ADSCs, which are replaced by connective and scar tissues [14,20]. This ADSC-led remodeling process may show fat grafting to be an ideal option for a long lasting, aesthetic treatment of cleft lip deformity.

ADSCs are numerous in fat samples of patients of all ages [6]. This should allow for an effective and aesthetic correction of lip deformity at any age as all patients have this abundant source of stem cells. Although the correction can be done at any time, Wu et al. showed increased angiogenic and osteogenic capabilities of infant ADSCs, which may demonstrate improved grafting when correction is performed at a younger age.

The ADSCs in the fat graft have other benefits as well. Not only can the fat be easily and safely collected from various sites in the body, but there is also minimal chance of rejection because the graft is autologous [2]. It also offers numerous benefits to angiogenesis, inflammation regulation, wound healing, and scar formation [4,5,7,8]. Favorable effects on scar healing may produce a more aesthetic appearance.

Parsaei et al. utilizes botulinum toxin A injections along with fat graft to further amplify the favorable effects of fat grafting on scar formation. Temporary paralysis of the orbicularis oris muscle after incision closure helped to eliminate tension on the site and minimize risks of hypertrophic scar formation [8]. Additionally, there is data supporting positive effects of botulinum toxin A on fat graft retention [23]. This may be due to enhancement of angiogenesis and adipogenesis at the graft site [24,25]. Reported graft reabsorption rates vary between 20% to 90% [23]. The mouth is a particularly mobile area that provides some challenges to fat grafting [8]. Fat stability and reabsorption are the major limiting factors to fat grafting and finding ways to correct these issues could make fat grafting the favorable method of repair [2]. Research thus far shows promise, but more human data on the utilizations of botulin toxin A in conjunction with fat grafting for cleft lip repair is needed to evaluate its effectiveness in long term graft retention.

All reports of fat grafting during primary repair and/or as a secondary repair showed favorable results with statistically significant increases in upper lip appearance, volume, and contour suggesting that autologous fat grafting can be utilized at either point of repair. Data on fat grafting during primary cleft lip repair is lacking; all favorable results are shown on grading scales used by reviewers [7,9]. However, a grading scale, especially one based upon aesthetics, relies on subjective opinions and may not be entirely reliable.

There were no reports of serious complications at graft or donor site; however Balkin et al. reported difficulty fitting nasal stents after the procedure [7,9]. While not a serious complication, infants are obligate nose breathers, and this may need to be considered when selecting an age for correction if grafting is performed during primary repair. The major limiting factors in these studies are small sample size and lack of documented long term follow-up, with the longest reported being around 60.2 months but the means being 24.7 months or greater than six months [7,9]. This may demonstrate usefulness in fat grafting during primary cleft repair early on, but a lack of long term follow-up fails to provide insight into how long these grafts survive and their appear in teenage and adult years.

Delayed fat grafting as a secondary repair has more data available. All articles showed statistically significant aesthetic outcomes in physical measurements and rating scales; however, results in patient satisfaction did not share unanimous results [2,10-13]. Jones et al. reported that 11 out of 18 patients completed satisfaction surveys that were unanimously positive. Baum et al. reported satisfaction of 10 out of 15 patients. Only one study reported complications, one reported hematoma, four reported patients with periumbilical pressure at donor site, and one report of long-term lip pain [13]. While these are the only reported complications, Baum et al. worked exclusively on patients with whistle deformity and data is not sufficient to determine if this issue is unique to fat grafting for correction of this issue. Baum et al. reported three patients and Jones et al. reported one who needed secondary fat grafting due to under correction. Despite the repeat grafts, all studies showed statistically significant increase in aesthetics of the lip post-grafting. Like primary repair, limitations lie in the small sample sizes, subjectivity of raters, and lack of long term follow-up.

Debate can be made as to the optimal time to perform fat grafting. Zellner et al. hypothesizes that the positive effects of ADSCs on wound healing may make fat grafting during primary repair favorable due to improved scar appearance and long-term results; however, data to support this claim is insufficient and requires long term follow-up with a larger sample size of patients who receive grafting during primary repair. The increased angiogenic effects of infantile ADSCs in comparison to adults support this view [6]. Arguments can be made to delay grafting until secondary repair as well. Delaying grafting to a secondary procedure allows the surgeon to more precisely identify deficient areas and deformities to correct with grafting [2].

There are some other important aspects that may affect graft retention as well. Scarred tissue proved more hostile to grafted fat, possibly due to compromised vascularity leading to decreased one year retention of the graft [17]. Obstacles affecting graft retention, such as previous scarring, are issues where overcorrection may be especially beneficial; however, further research into the optimal volume of overcorrection is lacking. There is not a currently agreed upon recommended time frame for re-evaluation of need and amount of subsequent grafting, but Denadai et al. suggests progressive retention of grafts during the first three months following repair with stabilization of the graft occurring at the 12-month mark. Reassessment of the need to perform additional grafting may be best performed during this time-frame.

Additionally, age plays a factor in retention as well. The benefits of infantile ADSCs were discussed previously, and this was further supported by a study showing pediatric patients with increased graft retention when compared against older patients [17]. Secondary correction during early childhood may be beneficial as it allows the surgeon to precisely treat the deformity while still taking advantage of young ADSCs. Further research into angiogenic abilities of ADSCs at different ages could prove useful in guiding this decision. There is limited data specifically on the pediatric population and fat grafting, with nothing available on long term follow-up into teenage and adult years to support effects of age or scars on graft survival and appearance over a long period. There is also some data in support of the utilization of microneedling pre-treatment to increase graft retention due to increases in vascularity at the site [19,26].

There are some other limitations in data existing as well. Small sample sizes, lack of long-term data, and subjectivity have been mentioned, but a lack of standardization of measurements may affect data as well. The subjectivity of raters is an issue that is difficult to correct, but the objective measurements are an aspect that can be standardized. There are a variety of ways to take objective measurements of lip volume. Some choose to utilize measurements based on pre- and postoperative photographs due to ease and cost efficiency; however, this may lead to greater risk of error when using a two-dimensional photo to evaluate a three-dimensional outcome. Imaging modalities such as MRI may offer a more accurate measurement; however, repeat MRIs are neither cost effective or convenient for the patient [27]. While Herold et al. performed their study on measuring of grafting in the breast, their recommendation for the utilization of three-dimensional surface scan may serve as a possible route of exploration to standardize measurement in a timely, accurate, and cost-effective manner.

In addition to lack of standardization in measurement, there is also no optimal technique in the literature. The Coleman technique is the most utilized, but without a standardization in technique the variety of donor sites, harvesting methods, refining methods, and injection methods may all play a role in graft appearance and survival [21]. Further research comparing different methods and techniques of extraction in a laboratory setting can guide choices to eventually discern an optimal technique in clinical practice.

## Conclusions

Autologous fat grafting is a safe and effective avenue for the treatment of deformity secondary to cleft lip repair. It is a minimally invasive method that provides positive aesthetic results. More data is needed regarding the long-term results of grafting during primary repair versus secondary repair. Based on current data, secondary repair may be preferential; however, both appear to be viable options. Patient and parent preferences to time of fat grafting and donor sites can be discussed to provide a positive result with minimal risk. More research is needed to investigate optimal techniques, most effective time to perform grafting, and the ideal volume to utilize for correction of the aesthetics post-cleft lip repair. 

Despite there being many areas where more data would be beneficial, longitudinal studies of lifetime graft retention may serve as the best evaluation of the efficacy of this method of deformity repair. Results to date have been promising. Due to an increase in lip volume, contour, and appearance, patient satisfaction is high, even in those with more severe deformities. As time progresses, and long-term results and graft retention become more observable, autologous fat grafts may become the standard of care when choosing a method to improve the volume and aesthetics of cleft lip deformity.
